# Historizando la eugenesia en Chile: saberes, agentes, prácticas médicas

**DOI:** 10.1590/S0104-59702025000100037

**Published:** 2025-09-15

**Authors:** Felipe Hidalgo Kawada

**Affiliations:** i Universidad Autónoma de Chile. Santiago – Chile fhid0279@uni.sydney.edu.au

En distintos lugares del mundo, se viven tiempos lúgubres e inciertos. En particular, los masivos procesos migratorios actuales están haciendo reflotar racismos, xenofobias, discriminaciones y violencias (Moreno, Wade, 2024). Latinoamérica es un ejemplo de estos fenómenos contemporáneos y Chile no ha sido la excepción (Tijoux, 2016). Es por eso que el libro que reseño en estas líneas ha llegado en un momento especialmente relevante.

La obra escrita por el historiador Marcelo Sánchez Delgado (2024) tiene como propósito analizar críticamente el proyecto eugenésico en Chile en un marco temporal que va desde fines del siglo XIX hasta 1941, con algunas referencias que sobrepasan esta temporalidad. En aquella época, la eugenesia se legitimó como una “ciencia” que buscaba mejorar la población humana mediante todos los medios biológicos y sociales disponibles. En este libro, se coloca el foco en las ideas eugénicas construidas, principalmente, en el campo de la medicina, estudiando sus actores y agentes, los saberes que hicieron circular, y los efectos que produjeron en el campo de la medicina y en el plano político y social. En gran parte, las fuentes consultadas provienen del campo de la medicina como, por ejemplo, la *Revista Médica de Chile*.

Este libro también resulta útil para un lector interesado en las trayectorias de diversos actores del campo de la salud que tuvieron distintos grados de relevancia, como, por ejemplo, Luis Vergara Flores, Augusto Orrego Luco, Florentino Caro, Germán Greve y Hugo Lea-Plaza analizados en el capítulo uno; Nicolás Palacios, Max Westenhöfer, Federico Johow y Juan Noé en el capítulo dos; Ottmar Wilhelm, Luis Bisquertt, Waldemar Coutts, Otto Aichel, Luis Tirapegui, Carlos Mönckeberg, y nuevamente Juan Noé en el capítulo tres; y Salvador Allende, Hans Betzhold y, de nuevo, la figura de Waldemar Coutts en el capítulo cuatro.

El libro está compuesto por una introducción, cuatro capítulos, y un epílogo. La introducción es interesante, pues comienza con una historia íntima, colocando a una persona como su madre como personaje principal. Sabemos que, en mayor o menor medida, las preguntas de investigación de las/os autores siempre tienen que ver con sus historias y biografías. Esto queda de manifiesto en el libro de Sánchez, lo que no solo explica su implicación emocional y biográfica con la temática, sino que también nos advierte de la importancia de atender a la eugenesia más allá de una temática meramente disciplinar, prestando atención a los diversos impactos que ella ha tenido en la sociedad chilena.

El primer capítulo comienza mapeando los escenarios sanitarios y sociales que permitieron la entrada de la eugenesia en el contexto chileno, analizando la recepción de las teorías de la degeneración, y los llamados a la acción y también, los efectos que produjo la eugenesia en la escena científica y sociopolítica. Se analiza, por ejemplo, cómo el alcoholismo fue leído desde estas perspectivas a partir de los trabajos del doctor Luis Vergara, quien se aventuraba a vincular el alcoholismo con la criminalidad, así como enfermedades nerviosas y mentales con las incipientes teorías de la degeneración. Por su parte, el segundo capítulo se dedica a analizar el proyecto eugenésico en Chile desde comienzos del siglo XX. En particular, explora los vínculos y cercanías que tuvo Chile con Alemania, en donde el campo médico chileno fue especialmente receptivo a las teorías e ideas de médicos y académicos alemanes. Este capítulo permite comprender cómo, en el nombre de la “ciencia”, los saberes psicológicos y psiquiátricos, la antropometría y biotipología, se amplificaron los antagonismos entre sujetos civilizados/primitivos o normales/anormales.

Temas variados encontramos en el tercer capítulo, en los que confluyen, por ejemplo, la creación de organizaciones como la Liga Chilena de Higiene Social o el Ministerio de Higiene, Asistencia y Previsión Social, o de nuevos medios escritos como el *Almanaque 18* y el *Boletín del Ministerio de Higiene*. Así también, vemos cómo un conjunto de temas variados en torno a la sexualidad, inteligencia, craneometría y educación física, entre otros, se vincularon al proyecto eugenésico entre 1917 y 1930. A modo personal, este capítulo podría tener una mayor claridad, que permita entender los vínculos entre cada uno de los temas expuestos y su trama general. A su vez, este capítulo podría reafirmar el esfuerzo y carácter enciclopédico del libro, visibilizando diversas aristas que pueden ser futuras líneas de trabajo para otras/os investigadoras/es.

Por su parte, el cuarto capítulo presenta las discusiones en torno a la idea de implementar la esterilización eugénica en Chile. Se muestran los diversos argumentos que circularon en torno a la idea de esterilización, la posición de la Iglesia en esta coyuntura política, y de las voces médicas que participaron en este debate como Waldemar Coutts, Salvador Allende y Alejandro Lipschütz.

El capítulo que actúa “a modo de Epílogo”, narra la historia en torno a la obra *El vuelo del genio*, del escultor chileno-alemán Tótila Albert, la cual fue utilizada en el edificio sede de la institución estatal Defensa de la Raza y Aprovechamiento de las Horas Libres, en 1941. Se examina el carácter peculiar y el distanciamiento que tuvo esta institución en comparación a los principios eugénicos foráneos, además de presentar aspectos biográficos de Tótila. Llamativamente, este es el único capítulo que agrega material gráfico, con dos fotografías. En ese sentido, hubiese sido interesante quizás agregar más registros visuales a lo largo del libro para que se entretejieran con la narrativa contada.

En conclusión, este libro constituye una importante contribución al campo de los estudios de la historia de la medicina, la ciencia y la salud en Chile. Además, nos permite observar desde el presente la vigencia y cierta continuidad de ideas y elementos planteados por la eugenesia del siglo XX en los escenarios actuales. En ese sentido, este libro aporta también, tal como señala Sánchez, a una tarea mayor: resignificar la vida y construir alternativas posibles.
